# Cross-Situational Learning with Bayesian Generative Models for Multimodal Category and Word Learning in Robots

**DOI:** 10.3389/fnbot.2017.00066

**Published:** 2017-12-19

**Authors:** Akira Taniguchi, Tadahiro Taniguchi, Angelo Cangelosi

**Affiliations:** ^1^Emergent Systems Laboratory, Ritsumeikan University, Kusatsu, Japan; ^2^The Centre for Robotics and Neural Systems, Plymouth University, Plymouth, United Kingdom

**Keywords:** Bayesian model, cross-situational learning, lexical acquisition, multimodal categorization, symbol grounding, word meaning

## Abstract

In this paper, we propose a Bayesian generative model that can form multiple categories based on each sensory-channel and can associate words with any of the four sensory-channels (action, position, object, and color). This paper focuses on cross-situational learning using the co-occurrence between words and information of sensory-channels in complex situations rather than conventional situations of cross-situational learning. We conducted a learning scenario using a simulator and a real humanoid iCub robot. In the scenario, a human tutor provided a sentence that describes an object of visual attention and an accompanying action to the robot. The scenario was set as follows: the number of words per sensory-channel was three or four, and the number of trials for learning was 20 and 40 for the simulator and 25 and 40 for the real robot. The experimental results showed that the proposed method was able to estimate the multiple categorizations and to learn the relationships between multiple sensory-channels and words accurately. In addition, we conducted an action generation task and an action description task based on word meanings learned in the cross-situational learning scenario. The experimental results showed that the robot could successfully use the word meanings learned by using the proposed method.

## 1 Introduction

This paper addresses the study of robotic learning of the word meanings inspired by the process of language acquisition of humans. We developed an unsupervised machine learning method to enable linguistic interaction between humans and robots. Human infants can acquire word meanings by estimating the relationships between multimodal information and words in a variety of situations. For example, if an infant grasps a green cup by hand, let us consider the way the parent describes the actions of the infant to the infant using a sentence such as “grasp green front cup.” In this case, the infant does not know the relationship between words and situations because it has not acquired the meanings of words. In other words, the infant cannot determine whether the word “green” indicates an action, an object, or a color. However, it is believed that the infant can learn that the word “green” represents the green color by observing the co-occurrence of the word “green” with objects of green color in various situations. This is known as cross-situational learning (CSL), which has been both studied in children (Smith et al., 2011) and modeled in simulated agents and robots (Fontanari et al., 2009). The CSL is related to the symbol grounding problem (Harnad, 1990), which is a challenging and significant issue in robotics.

The generalization ability and the robustness of observation noise to process situations that have never been experienced are important in cognitive robotics. The study of language acquisition by infants led to the proposal of a hypothesis of taxonomic bias (Markman and Hutchinson, 1984) that infants tend to understand a word as the name of a category to which the target object belongs rather than a proper noun. This hypothesis could also be considered to play an important role in CSL. In this study, we assume that words are associated with categories based on taxonomic bias. By associating words with categories, it becomes possible for a human to generalize and process words. Therefore, humans can use words for communication in new situations. To develop this ability, the robot needs to form categories from observation information autonomously. We develop this ability by categorization based on the Bayesian generative model. Another hypothesis regarding the lexical acquisition by an infant was mutual exclusivity bias (constraint) (Markman and Wachtel, 1988). In studies on lexical acquisition, this hypothesis was considered to be particularly important for CSL (Twomey et al., 2016). Mutual exclusivity bias assumes that the infant considers the name of an object to correspond to one particular category only. In other words, multiple categories do not correspond to that word simultaneously. In Imai and Mazuka (2007), it was suggested that once an infant decides whether a word refers to the name of an object or a substance, the same word is not applied across the ontological distinction such as objects and substances. In this study, we extend the mutual exclusivity constraint to the CSL problem in complex situations. We aim to develop a novel method that can acquire knowledge of multiple categories and word meanings simultaneously. In addition, we verify whether the effect of mutual exclusivity is biased toward lexical acquisition by constructing a model assuming different constraints.

In addition, humans can perform the instructed action using acquired knowledge. For example, the parent places some objects in front of an infant and speaks “grasp green right ball” to the infant. In this case, the infant can use the acquired word meanings to select the green ball to the right of some objects and perform the action of grasping. Furthermore, humans can explain self-action with the sentence using the acquired knowledge. For example, if the infant knows the word meanings after grasping a blue box in front of it, the infant can speak “grasp blue front box” to another person. Understanding instructions and describing situations are crucial problems that are also required to build a cognitive robot.

In this paper, the goal is to develop an unsupervised machine-learning method for learning the relationships between words and the four sensory-channels (action, object, color, and position) from the robot’s experience of observed sentences describing object manipulation scenes. In the above example, sentences containing four words for four sensory-channels are shown. However, in the scenario described in this study, sentences of less than four words are allowed. In addition, the position sensory-channel corresponds to the original position of the object. In other words, we assume that the environment is static. We assume that the robot can recognize spoken words without errors, as this work focuses specifically on (1) the categorization for each sensory-channel, (2) the learning of relationships between words and sensory-channels, and (3) the grounding of words in multiple categories. In addition, we demonstrate whether the robot can carry out its actions and the sentence description of its action by conducting experiments using the CSL results. The main contributions of this paper are as follows:

- We proposed an unsupervised machine-learning method based on a Bayesian generative model that makes it possible to learn word meanings, i.e., the relationships between words and categories, from complex situations.
- We demonstrated that word meanings learned by using the proposed method are effective for generating an action and description of a situation.

The remainder of this paper is organized as follows. In Section 2, we discuss previous studies on lexical acquisition by a robot and CSL that are relevant to our study. In Section 3, we present a proposed Bayesian generative model for CSL. In Sections 4 and 5, we discuss the effectiveness of the proposed method in terms of three tasks, i.e., cross-situational learning, action generation, and an action description task, in a simulation and a real environment, respectively. Section 6 concludes the paper.

## 2 Related Work

### 2.1 Lexical Acquisition by Robot

Studies of language acquisition also constitute a constructive approach to the human developmental process (Cangelosi and Schlesinger, 2015), the language grounding (Steels and Hild, 2012), and the symbol emergence (Taniguchi et al., 2016c). One approach to studying language acquisition focuses on the estimation of phonemes and words from speech signals (Goldwater et al., 2009; Heymann et al., 2014; Taniguchi et al., 2016d). However, these studies used only continuous speech signals without using co-occurrence based on other sensor information, e.g., visual, tactile, and proprioceptive information. Therefore, the robot was not required to understand the meaning of words. Yet, it is important for a robot to understand word meanings, i.e., grounding the meanings to words, for human–robot interaction (HRI).

Roy and Pentland (2002) proposed a computational model by which a robot could learn the names of objects from images of the object and natural infant-directed speech. Their model could perform speech segmentation, lexical acquisition, and visual categorization. Hörnstein et al. (2010) proposed a method based on pattern recognition and hierarchical clustering that mimics a human infant to enable a humanoid robot to acquire language. Their method allowed the robot to acquire phonemes and words from visual and auditory information through interaction with the human. Nakamura et al. (2011a,b) proposed multimodal latent Dirichlet allocation (MLDA) and a multimodal hierarchical Dirichlet process (MHDP) that enables the categorization of objects from multimodal information, i.e., visual, auditory, haptic, and word information. Their methods enabled more accurate object categorization by using multimodal information. Taniguchi et al. (2016a) proposed a method for simultaneous estimation of self-positions and words from noisy sensory information and an uttered word. Their method integrated ambiguous speech recognition results with the self-localization method for learning spatial concepts. However, Taniguchi et al. (2016a) assumed that the name of a place would be learned from an uttered word. Taniguchi et al. (2016b) proposed a nonparametric Bayesian spatial concept acquisition method (SpCoA) based on place categorization and unsupervised word segmentation. SpCoA could acquire the names of places from spoken sentences including multiple words. In the above studies, the robot was taught to focus on one target, e.g., an object or a place, by a tutor using one word or one sentence. However, considering a more realistic problem, the robot needs to know which event in a complicated situation is associated with which word in the sentence. The CSL, which is extended from the aforementioned studies on the lexical acquisition, is a more difficult and important problem in robotics in comparison. Our research concerns the CSL problem because of its importance in relation to the lexical acquisition by a robot.

### 2.2 Cross-Situational Learning

#### 2.2.1 Conventional Cross-Situational Learning Studies

Frank et al. (2007, 2009) proposed a Bayesian model that unifies statistical and intentional approaches to cross-situational word learning. They conducted basic CSL experiments with the purpose of teaching an object name. In addition, they discussed that the effectiveness of mutual exclusivity for CSL in probabilistic models. Fontanari et al. (2009) performed object-word mapping from the co-occurrence between objects and words by using a method based on neural modeling fields (NMF). In “modi” experiments using iCub, their findings were similar to those reported by Smith and Samuelson (2010). The abovementioned studies are CSL studies that were inspired by studies based on experiments with human infants. These studies assumed a simple situation such as learning the relationship between objects and words as the early stage of CSL. However, the real environment is varied and more complex. In this study, we focus on the problem of CSL in utterances including multiple words and observations from multiple sensory-channels.

#### 2.2.2 Probabilistic Models

Qu and Chai (2008, 2010) proposed a learning method that automatically acquires novel words for an interactive system. They focused on the co-occurrence between word-sequences and entity-sequences tracked by eye-gaze in lexical acquisition. Qu and Chai’s method, which is based on the IBM-translation model (Brown et al., 1993), estimates the word-entity association probability. However, their studies did not result in perfect unsupervised lexical acquisition because they used domain knowledge based on WordNet. Matuszek et al. (2012) presented a joint model of language and perception for grounded attribute learning. This model enables the identification of which novel words correspond to color, shape, or no attribute at all. Celikkanat et al. (2014) proposed an unsupervised learning method based on latent Dirichlet allocation (LDA) that allows many-to-many relationships between objects and contexts. Their method was able to predict the context from the observation information and plan the action using learned contexts. Chen et al. (2016) proposed an active learning method for cross-situational learning of object-word association. In experiments, they showed that LDA was more effective than non-negative matrix factorization (NMF). However, they did not perform any HRI experiment using the learned language. In our study, we perform experiments that use word meanings learned in CSL to generate an action and explain a current situation.

#### 2.2.3 Neural Network Models

Yamada et al. (2015, 2016) proposed a learning method based on a stochastic continuous-time recurrent neural network (CTRNN) and a multiple time-scales recurrent neural network (MTRNN). They showed that the learned network formed an attractor structure representing both the relationships between words and action and the temporal pattern of the task. Stramandinoli et al. (2017) proposed partially recurrent neural networks (P-RNNs) for learning the relationships between motor primitives and objects. Zhong et al. (2017) proposed multiple time-scales gated recurrent units (MTGRU) inspired by MTRNN and long short-term memory (LSTM) (Hochreiter and Schmidhuber, 1997). They showed that the MTGRU could learn long-term dependencies in large-dimensional multimodal datasets by conducting multimodal interaction experiments using iCub. The learning results of the above studies using neural networks (NNs) are difficult to interpret because time-series data is mapped to continuous latent space. These studies implicitly associate words with objects and actions. Generally, NN methods require a massive amount of learning data in many cases. On the other hand, the learning result is easier to interpret when Bayesian methods rather than NN methods are used. In addition, Bayesian methods require less data to learn efficiently. We propose a Bayesian generative model that can perform CSL, including action learning.

#### 2.2.4 Robot-to-Robot Interaction

Spranger (2015) and Spranger and Steels (2015) proposed a method for the co-acquisition of semantics and syntax in the spatial language. The experimental results showed that the robot could acquire spatial grammar and categories related to spatial direction. Heath et al. (2016) implemented mobile robots (Lingodroids) capable of learning a lexicon through robot-to-robot interaction. They used two robots equipped with different sensors and simultaneous localization and mapping (SLAM) algorithms. These studies reported that the robots created their lexicons in relation to places and the distance in terms of time. However, these studies did not consider lexical acquisition by HRI. We consider HRI to be necessary to enable a robot to learn human language.

#### 2.2.5 Multimodal Categorization and Word Learning

Attamimi et al. (2016) proposed multilayered MLDA (mMLDA) that hierarchically integrates multiple MLDAs as an extension of Nakamura et al. (2011a). They performed an estimation of the relationships among words and multiple concepts by weighting the learned words according to their mutual information as a post-processing step. In their model, the same uttered words are generated from three kinds of concepts, i.e., this model has three variables for same word information in different concepts. We consider this to be an unnatural assumption as the generative model for generating words. However, in our proposed model, we assume that the uttered words are generated from one variable. We consider our proposed model to involve a more natural assumption than Attamimi’s model. In addition, their study did not use data that were autonomously obtained by the robot. In Attamimi et al. (2016), it was not possible for the robot to learn the relationships between self-actions and words because human motions obtained by the motion capture system based on Kinect and a wearable sensor device attached to a human were used as action data. In our study, the robot learns the action category based on subjective self-action. Therefore, the robot can perform a learned action based on a sentence of human speech. In this paper, we focus on complicated CSL problems arising from situations with multiple objects and sentences including words related to various sensory-channels such as the names, position, and color of objects, and the action carried out on the object.

## 3 Multichannel Categorizations and Learning the Meaning of Words

We propose a Bayesian generative model for cross-situational learning. The proposed method can estimate categories of multiple sensory-channels and the relationships between words and sensory-channels simultaneously.

### 3.1 Overview of the Scenario and Assumptions

**Figure 1:**
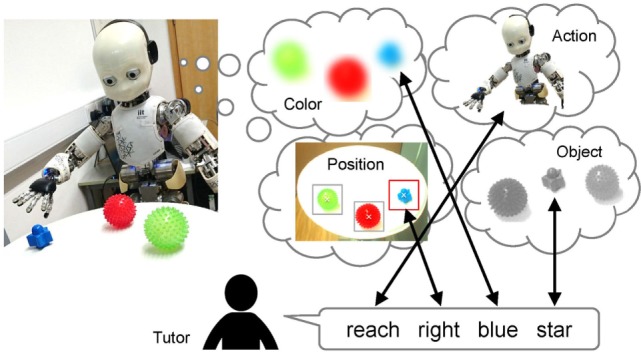
Overview of the cross-situational learning scenario as the focus of this study; the robot obtains multimodal information from multiple sensory-channels in a situation and estimates the relationships between words and sensory-channels.

Here, we provide an overview of the scenario on which we focus and some of the assumptions in this study. Figure 1 shows an overview of the scenario. The robot does not have any specific knowledge of objects, but it can recognize that objects exist on the table, i.e., the robot can segment the object and then extract the features of the segmented object. In addition, we assume that the robot can recognize the sentence uttered by the tutor without error. The training procedure consists of the following steps:

1. The robot is in front of the table on which the objects are placed. Multiple objects are placed separately on the table.
2. The robot selects an object from the objects on the table. The robot pays visual attention to a selected object, and then, exerts an action on the selected object, e.g., “grasp,” “touch,” “reach,” and “look-at.”
3. The human tutor utters a sentence including words about the object at which the robot is gazing and also a word about the action performed by the robot, e.g., “grasp front green cup.”
4. The robot obtains multimodal information regarding all objects on the table in the current situation, e.g., the object features, positions, colors, and self-action. The robot processes the sentence to discover the meanings of the words.

This process (steps 1–4) is carried out many times in different situations.

We assume that the robot does not know the relationships between the words and sensory-channels in advance. This study does not consider grammar, i.e., a unigram language model is assumed. The robot learns word meanings and multiple categories by using visual, tactile, and proprioceptive information, as well as words.

In this study, we consider two-level cross-situational learning (CSL-I and II). The first level (CSL-I) is the selection of an object related to a tutor utterance from multiple objects on the table. The second level (CSL-II) is the selection of the relationship between the specific word in the sentence and a sensory-channel in the multimodal information. In the first level, we assume joint attention. Tomasello and Farrar (1986) showed that the utterance referring to the object on which the child’s attention was already focused is more effective in language acquisition. The above scenario enables the tutor to identify the object of attention, i.e., the object at which the robot is gazing. Furthermore, we assume that the robot considers the tutor to be speaking a sentence concerning the object of attention. This assumption of joint attention can avoid the problem of the selection of an object. The second level is the main problem in this study. Many previous studies on CSL-I have been reported (Frank et al., 2007, 2009; Fontanari et al., 2009; Morse et al., 2010); however, there are not the case for studies on CSL-II. The study discussed in this paper focused on solving the crucial problem of CSL-II.

In this study, we assume a two-level mutual exclusivity constraint (Markman and Wachtel, 1988) (MEC-I and II) regarding the selection of the sensory-channel. The first level (MEC-I) is the mutual exclusivity of sensory-channels with a word, i.e., one word is allocated to one category in one sensory-channel. The second level (MEC-II) is the mutual exclusivity between sensory-channels indicated by words, i.e., one word related to each sensory-channel is spoken only once in a sentence (or is not spoken). MEC-II is a stronger constraint than MEC-I. The proposed method can include both levels of mutual exclusivity.

### 3.2 Generative Model and Graphical Model

**Figure 2:**
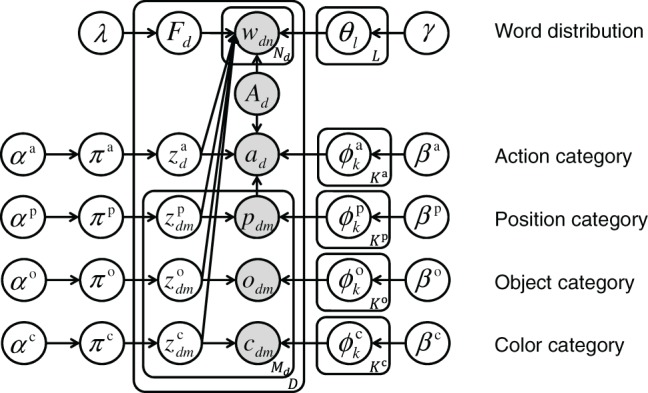
Proposed graphical model for multichannel categorizations and for learning word meaning; the action, position, color, and object categories are represented by a component in Gaussian mixture models (GMMs). A word distribution is related to a category on GMMs. Gray nodes represent observed variables. Each variable is explained in the description of the generative model in Section 3.2.

The generative model of the proposed method is defined as equations (1–10). Figure 2 shows a graphical model representing the probabilistic dependencies between variables of the generative model. Basically, the categorization for each sensory-channel is based on the Gaussian mixture model (GMM). In this model, the probability distribution of words is represented by the categorical distribution. The categorization of words in sentences is similar to that of LDA. The latent variable of a word shares the latent variable of any one of the sensory-channels in GMMs, signifying that a word and a category in a particular sensory-channel are generated from the same latent variable.

We describe the generative model as follows:

(1) $$F_{d}∼\text{Unif}\left(\lambda \right)$$

(2) $$\theta_{l}∼\text{Dir}\left(\gamma \right)$$

(3) π∼GEM(α)

(4) $$z_{\mathrm{dm}}∼\text{Cat}\left(\pi \right)$$

(5) $$w_{\mathrm{dn}}∼\text{Cat}\;\left(\theta_{l=\left(F_{\mathrm{dn}},z_{dA_{d}}^{F_{\mathrm{dn}}}\right)}\right)$$

(6) $$\phi_{k}∼\text{GIW}\left(\beta \right)$$

(7) $$o_{\mathrm{dm}}∼\text{Gauss}\left(\phi_{z_{\mathrm{dm}}^{\text{o}}}^{\text{o}}\right)$$

(8) $$c_{\mathrm{dm}}∼\text{Gauss}\left(\phi_{z_{\mathrm{dm}}^{\text{c}}}^{\text{c}}\right)$$

(9) $$p_{\mathrm{dm}}∼\text{Gauss}\left(\phi_{z_{\mathrm{dm}}^{\text{p}}}^{\text{p}}\right)$$

(10) $$a_{d}∼\text{Gauss}\left(\phi_{z_{d}^{\text{a}}}^{{\text{a}}^{\prime }}\right),$$

where the discrete uniform distribution is denoted as Unif(⋅), the categorical distribution is denoted as Cat(⋅), the Dirichlet distribution is denoted as Dir(⋅), the stick-breaking process (SBP) (Sethuraman, 1994) is denoted as GEM(⋅), the Gaussian-inverse-Wishart distribution is denoted as GIW(⋅), and the multivariate Gaussian distribution is denoted as Gauss(⋅). See Murphy (2012) for specific formulas of the above probability distributions. In this paper, variables omitting superscript represent general notation, e.g., *π* ∈ {*π*} = {*π*^a^, *π*^p^, *π*^o^, *π*^c^}, and variables omitting subscripts represent collective notation, e.g., *F* = {*F*_1_, *F*_2_, … *F_D_*}. The number of trials is *D*. The number of objects on the table is *M_d_* in the *d*-th trial. The number of words in the sentence is *N_d_* in the *d*-th trial. The *n*-th word in the *d*-th trial is denoted as *w_dn_*, which is represented by the bag-of-words (BoW). The model allows sentences containing zero to four words. The model associates the word distributions *θ* with categories *z_dm_* on four sensory-channels, namely, the action *a_d_*, the position *p_dm_* of the object on the table, the object feature *o_dm_*, and the object color *c_dm_*. In this study, we define the action *a_d_* as a static action feature, i.e., proprioceptive and tactile features, when the robot completes an action. An index of the object of attention selected by the robot from among the multiple objects on the table is denoted as A*_d_* = *m*. The sequence representing the respective sensory-channels associated with each word in the sentence is denoted as *F_d_*, e.g., *F_d_* = (a, p, c, o). The number of categories for each sensory-channel is *K*. An index of the word distribution is denoted as *l*. The set of all the word distributions is denoted as $\theta =\left\{\theta_{l=\left(F_{\mathrm{dn}},z_{\mathrm{dm}}^{F_{\mathrm{dn}}}\right)}|F_{\mathrm{dn}}\in \left\{\text{o},\text{c},\text{p},\text{a}\right\},z_{\mathrm{dm}}^{F_{\mathrm{dn}}}\in \left\{1,2,\;\ldots \;,K^{F_{\mathrm{dn}}}\right\}\right\}$. The index of the category of the sensory-channel *F_dn_* and the object *A_d_* is denoted as $z_{dA_{d}}^{F_{\mathrm{dn}}}$. Then, the number of word distributions *L* is the sum of the number of categories of all the sensory-channels, i.e., $L=K^{a}+K^{p}+K^{o}+K^{c}$. The action category $\phi_{k}^{\text{a}}$, the position category $\phi_{k}^{\text{p}}$, the object category $\phi_{k}^{\text{o}}$, and the color category $\phi_{k}^{\text{c}}$ are represented by a Gaussian distribution. The mean vector and the covariance matrix of the Gaussian distribution are denoted as *ϕ*_k_ = {*μ_k_*, Σ*_k_*}. We define $\phi_{z_{d}^{\text{a}}}^{{\text{a}}^{\prime }}$ as the parameter of the Gaussian distribution that added the object position $p_{dA_{d}}$ to the element of the mean vector representing the relative coordinates between the hand position and the target position. The position information of the object A*_d_* = *m* is denoted as $p_{dA_{d}}$. Therefore, $\phi_{z_{d}^{\text{a}}}^{{\text{a}}^{\prime }}$ is the parameter obtained by converting the target hand position to the absolute coordinate system based on $\phi_{z_{d}^{\text{a}}}^{\text{a}}$ (the parameter of the action category represented in the relative coordinate system) and $p_{dA_{d}}$ (the position of the object of attention). The mixture weights of the categories for each sensory-channel are denoted as *π*^a^, *π*^p^, *π*^o^, and *π*^c^. The hyperparameter *λ* of the uniform distribution, i.e., equation (1), has the mutual exclusivity constraint that determines that each sensory-channel is represented only once in each sentence. The hyperparameter of the mixture weights *π* is denoted as *α*. The hyperparameter of the Gaussian-inverse-Wishart distribution is denoted as *β* = {*m*_0_, *κ*_0_, *V*_0_, *v*_0_}. The hyperparameter of the Dirichlet distribution is denoted as *γ*. Italic notation (*a*, *p*, *o*, *c*) represents observation variables, ordinary notation used as a superscript (a, p, o, c) represents sensory-channels.

The robot needs to estimate the number of categories based on experience because the robot cannot have previous knowledge about categories. The proposed method can learn an appropriate number of categories, depending on the collected data, by using a nonparametric Bayesian approach. Specifically, this method uses the SBP, a method based on the Dirichlet process. Therefore, this method can consider theoretically infinite numbers *K*^a^, *K*^p^, *K*^o^, and *K*^c^. In this paper, we approximate the values of parameters representing the number of categories *K*^a^, *K*^p^, *K*^o^, and *K*^c^ by assigning sufficiently large values, i.e., a weak-limit approximation (Fox et al., 2011).

### 3.3 Learning Algorithm

This model estimates parameters representing multiple categories, word distribution, the relationships between the word and the sensory-channel as input for the object features, positions, colors, robot actions, and the sentences spoken by a tutor. The model parameters and latent variables of the proposed method are estimated from the following joint posterior distribution by Gibbs sampling:

(11) Θ,Z∼p(Θ,Z|X,H),

where the set of model parameters is denoted as Θ = {{*π*}, {*ϕ*}, *θ*}, the set of latent variables is denoted as Z = {{*z*}, *F*}, the set of observation variables is denoted as *X* = {*a*, *p*, *o*, *c*, *w*, *A*}, and the set of hyperparameters of the model is denoted as *H* = {{*α*}, {*β*}, *λ*, *γ*}.

The learning algorithm is obtained by repeatedly sampling the conditional posterior distributions for each parameter. The Dirichlet and GIW distributions are conjugate prior distributions for the categorical and Gaussian distributions, respectively (Murphy, 2012). Therefore, the conditional posterior distributions can be determined analytically. Algorithm 1 shows the pseudo-code for the learning procedure. The initial values of the model parameters can be set arbitrarily in accordance with a condition. The following is the conditional posterior distribution of each element used for performing Gibbs sampling.

**Algorithm 1:** Learning algorithm based on Gibbs sampling.

1: **procedure** Gibbs_Sampling (*a, p, o, c, w, A*)
2: Setting of hyperparameters {*α*}, {*β*}, *λ*, *γ*
3: Initialization of parameters and latent variables {*π*}, {*ϕ*}, *θ*, {*z*}, *F*
4: **for** *j* = 1 to *iteration*_*number* **do**
5: *π*^o^ ∼ Dir(*π*^o^ | *z*^o^, *α*^o^) // equation (12)
6: *π*^c^ ∼ Dir(*π*^c^| *z*^c^, *α*^c^) // equation (13)
7: *π*^p^ ∼ Dir(*π*^p^| *z*^p^, *α*^p^) // equation (14)
8: *π*^a^ ∼ Dir(*π*^a^| *z*^a^, *α*^a^) // equation (15)
9: **for** *k* = 1 to *K*^o^ **do**
10: $\phi_{k}^{\text{o}}∼\text{GIW}\left(\phi_{k}^{\text{o}}|o_{k},\beta^{\text{o}}\right)$ // equation (16)
11: **end for**
12: **for** *k* = 1 to *K*^c^ **do**
13: $\phi_{k}^{\text{c}}∼\text{GIW}\left(\phi_{k}^{\text{c}}|c_{k},\beta^{\text{c}}\right)$ // equation (17)
14: **end for**
15: **for** *k* = 1 to *K*^p^ **do**
16: $\phi_{k}^{\text{p}}∼\text{GIW}\left(\phi_{k}^{\text{p}}|p_{k},\beta^{\text{p}}\right)$ // equation (18)
17: **end for**
18: **for** *k* = 1 to *K*^a^ **do**
19: $\phi_{k}^{\text{a}}∼\text{GIW}\left(\phi_{k}^{\text{a}}|a_{k}^{\prime },\beta^{\text{a}}\right)$ // equation (19)
20: **end for**
21: **for** $l=\left(F_{\mathrm{dn}},z_{{\mathrm{dA}}_{d}}^{F_{\mathrm{dn}}}\right)\;\text{in}\;\left\{\left(F_{\mathrm{dn}},z_{\mathrm{dm}}^{F_{\mathrm{dn}}}\right)|\;F_{\mathrm{dn}}\in \left\{\text{o},\text{c},\text{p},\text{a}\right\},\right.$ $\left.z_{\mathrm{dm}}^{F_{\mathrm{dn}}}\in \left\{\text{1},\text{2},\;\ldots \;,\;K^{F_{\mathrm{dn}}}\right\}\right\}$ **do**
22: *θ*_l_ ∼ Dir(θ_l_ | *w_l_*, *γ*) // equation (20)
23: **end for**
24: **for** *d* = 1 to *D* **do**
25: **for** *m* = 1 to *M_d_* **do**
26: $z_{\mathrm{dm}}^{\text{o}}∼\prod_{n=1}^{N_{d}}\text{ Cat}\;\left(w_{\mathrm{dn}}|\theta_{l=\left(F_{\mathrm{dn}},z_{dA_{d}}^{F_{\mathrm{dn}}}\right)}\right)\;\text{Gauss}\;\left(o_{\mathrm{dm}}|\phi_{z_{\mathrm{dm}}^{\text{o}}}^{\text{o}}\right)\;\text{Cat}\left(z_{\mathrm{dm}}^{\text{o}}|\pi^{\text{o}}\right)$ // equation (21)
27: $z_{\mathrm{dm}}^{\text{c}}∼\prod_{n=1}^{N_{d}}\text{ Cat}\;\left(w_{\mathrm{dn}}|\theta_{l=\left(F_{\mathrm{dn}},z_{dA_{d}}^{F_{\mathrm{dn}}}\right)}\right)\;\text{Gauss}\;\left(c_{\mathrm{dm}}|\phi_{z_{\mathrm{dm}}^{\text{c}}}^{\text{c}}\right)\;\text{Cat}\left(z_{\mathrm{dm}}^{\text{c}}|\pi^{\text{c}}\right)$ // equation (22)
28: $z_{\mathrm{dm}}^{\text{p}}∼\prod_{n=1}^{N_{d}}\text{ Cat}\;\left(w_{\mathrm{dn}}|\theta_{l=\left(F_{\mathrm{dn}},z_{dA_{d}}^{F_{\mathrm{dn}}}\right)}\right)\;\text{Gauss}\;\left(p_{\mathrm{dm}}|\phi_{z_{\mathrm{dm}}^{\text{p}}}^{\text{p}}\right)\;\text{Cat}\left(z_{\mathrm{dm}}^{\text{p}}|\pi^{\text{p}}\right)$ // equation (23)
29: **end for**
30: $z_{d}^{\text{a}}∼\prod_{n=\text{1}}^{N_{d}}\text{ Cat}\;\left(w_{\mathrm{dn}}|\theta_{l=\left(F_{\mathrm{dn}},z_{dA_{d}}^{F_{\mathrm{dn}}}\right)}\right)\;\text{Gauss}\;\left(a_{d}|\phi_{z_{d}^{\text{a}}}^{{\text{a}}^{\prime }}\right)\;\text{Cat}\left(z_{d}^{\text{a}}|\pi^{\text{a}}\right)$ // equation (24)
31: $F_{d}∼\prod_{n=1}^{N_{d}}\text{ Cat}\;\left(w_{\mathrm{dn}}|\theta_{l=\left(F_{\mathrm{dn}},z_{dA_{d}}^{F_{\mathrm{dn}}}\right)}\right)\;\text{Unif}\left(F_{d}|\lambda \right)$ // equation (25)
32: **end for**
33: **end for**
34: **return** {*π*}, {*ϕ*}, *θ*, {*z*}, *F*
35: **end procedure**

A parameter *π*^o^ of categorical distribution representing the mixture weight of an object category is sampled as follows:

(12) $$\pi^{\text{o}}∼p\left(\pi^{\text{o}}|z^{\text{o}},\alpha^{\text{o}}\right)\propto \overset{D}{\underset{d=1}{\prod }}\overset{M_{d}}{\underset{m=1}{\prod }}\text{ Cat}\left(z_{\mathrm{dm}}^{\text{o}}|\pi^{\text{o}}\right)\text{Dir}\left(\pi^{\text{o}}|\alpha^{\text{o}}\right)\propto \text{Dir}\left(\pi^{\text{o}}|z^{\text{o}},\alpha^{\text{o}}\right),$$

where *z*^o^ denotes the set of all the latent variables of an object category. A parameter *π*^c^ of categorical distribution representing the mixture weight of the color category is sampled as follows:

(13) $$\pi^{\text{c}}∼p\left(\pi^{\text{c}}|z^{\text{c}},\alpha^{\text{c}}\right)\propto \overset{D}{\underset{d=1}{\prod }}\;\overset{M_{d}}{\underset{m=1}{\prod }}\text{ Cat}\left(z_{\mathrm{dm}}^{\text{c}}|\pi^{\text{c}}\right)\text{Dir}\left(\pi^{\text{c}}|\alpha^{\text{c}}\right)\;\propto \text{Dir}\left(\pi^{\text{c}}|z^{\text{c}},\alpha^{\text{c}}\right),$$

where *z*^c^ denotes a set of all the latent variables of the color category. A parameter *π*^p^ of the categorical distribution representing the mixture weight of the position category is sampled as follows:

(14) $$\pi^{p}∼p\left(\pi^{\text{p}}|z^{\text{p}},\alpha^{\text{p}}\right)\propto \overset{D}{\underset{d=1}{\prod }}\;\overset{M_{d}}{\underset{m=1}{\prod }}\text{ Cat}\left(z_{\mathrm{dm}}^{\text{p}}|\pi^{\text{p}}\right)\;\text{Dir}\left(\pi^{\text{p}}|\alpha^{\text{p}}\right)\;\propto \text{Dir}\left(\pi^{\text{p}}|z^{\text{p}},\alpha^{\text{p}}\right),$$

where *z*^p^ denotes the set of all the latent variables of the position category. A parameter *π*^a^ of the categorical distribution representing the mixture weight of the action category is sampled as follows:

(15) $$\pi^{\text{a}}∼p\left(\pi^{\text{a}}|z^{\text{a}},\alpha^{\text{a}}\right)\propto \;\overset{D}{\underset{d=1}{\prod }}\;\text{Cat}\left(z_{d}^{\text{a}}|\pi^{\text{a}}\right)\text{Dir}\left(\pi^{\text{a}}|\alpha^{\text{a}}\right)\propto \text{Dir}\left(\pi^{\text{a}}|z^{\text{a}},\alpha^{\text{a}}\right),$$

where *z*^a^ denotes a set of all the latent variables of the action category. A parameter $\phi_{k}^{\text{o}}$ of the Gaussian distribution of the object category is sampled for each *k* ∈ {1, 2, … , *K*^o^} as follows:

(16) $$\phi_{k}^{\text{o}}∼p\left(\phi_{k}^{\text{o}}|z^{\text{o}},o,\beta^{\text{o}}\right)\propto \overset{D}{\underset{d=1}{\prod }}\;\overset{M_{d}}{\underset{m=1}{\prod }}\text{ Gauss}\left(o_{\mathrm{dm}}|\phi_{k}^{\text{o}}\right)\;\text{GIW}\left(\phi_{k}^{\text{o}}|\beta^{\text{o}}\right)\;\propto \text{GIW}\left(\phi_{k}^{\text{o}}|o_{k},\beta^{\text{o}}\right),$$

where *o_k_* denotes a set of all the object features of the object category $z_{\mathrm{dm}}^{\text{o}}=k$ in *m* ∈ {1, 2, … , *M_d_*} and *d* ∈ {1, 2, … , *D*}. A parameter $\phi_{k}^{\text{c}}$ of the Gaussian distribution of the color category is sampled for each *k* ∈ {1, 2, … , *K*^c^} as follows:

(17) $$\phi_{k}^{\text{c}}∼p\left(\phi_{k}^{\text{c}}|z^{\text{c}},c,\beta^{\text{c}}\right)\propto \overset{D}{\underset{d=1}{\prod }}\;\overset{M_{d}}{\underset{m=1}{\prod }}\text{ Gauss}\left(c_{\mathrm{dm}}|\phi_{k}^{\text{c}}\right)\;\text{GIW}\left(\phi_{k}^{\text{c}}|\beta^{\text{c}}\right)\;\propto \text{GIW}\left(\phi_{k}^{\text{c}}|c_{k},\beta^{\text{c}}\right),$$

where *c_k_* denotes the set of all the color features of the color category $z_{\mathrm{dm}}^{\text{c}}=k$ in *m* ∈ {1, 2, … , *M_d_*} and *d* ∈ {1, 2, … , *D*}. A parameter $\phi_{k}^{\text{p}}$ of the Gaussian distribution of the position category is sampled for each *k* ∈ {1, 2, … , *K*^P^} as follows:

(18) $$\phi_{k}^{\text{p}}∼p\left(\phi_{k}^{\text{p}}|z^{\text{p}},p,\beta^{\text{p}}\right)\propto \overset{D}{\underset{d=1}{\prod }}\;\overset{M_{d}}{\underset{m=1}{\prod }}\text{ Gauss}\left(p_{\mathrm{dm}}|\phi_{k}^{\text{p}}\right)\;\text{GIW}\left(\phi_{k}^{\text{p}}|\beta^{\text{p}}\right)\;\propto \text{GIW}\left(\phi_{k}^{\text{p}}|p_{k},\beta^{\text{p}}\right),$$

where *p_k_* denotes the set of all the position information of the position category $z_{\mathrm{dm}}^{\text{p}}=k$ in *m* ∈ {1, 2, … , *M_d_*} and *d* ∈ {1, 2, … , *D*}. A parameter $\phi_{k}^{\text{a}}$ of the Gaussian distribution of the action category is sampled for each *k* ∈ {1, 2, … , *K*^a^} as follows:

(19) $$\phi_{k}^{\text{a}}∼p\left(\phi_{k}^{\text{a}}|z^{\text{a}},a,p,A,\beta^{\text{a}}\right)\propto \overset{D}{\underset{d=1}{\prod }}\text{ Gauss}\left(a_{d}^{\prime }|\phi_{k}^{\text{a}}\right)\;\text{GIW}\left(\phi_{k}^{\text{a}}|\beta^{\text{a}}\right)\;\propto \text{GIW}\left(\phi_{k}^{\text{a}}|a_{k}^{\prime },\beta^{\text{a}}\right),$$

where *a* denotes the set of all the action information, *p* denotes the set of all the position information, and *A* denotes the set of all the attention information. The element representing the relative coordinates of the hand of ${a^{\prime }}_{d}$ is calculated by the element representing the absolute coordinates of the hand of *a*, the object positions *p*, and the attention information *A*. The set of all the action information of the action category $z_{d}^{\text{a}}=k$ in *d* ∈ {1, 2, … , *D*} is denoted as $a_{k}^{\prime }$. A parameter θ_l_ of the word probability distribution is sampled for each $l\in \left\{\left(F_{\mathrm{dn}},z_{\mathrm{dm}}^{F_{\mathrm{dn}}}\right)|F_{\mathrm{dn}}\in \left\{\text{o},\text{c},\text{p},\text{a}\right\},z_{\mathrm{dm}}^{F_{\mathrm{dn}}}\in \left\{1,2,\;\ldots \;,\;K^{F_{\mathrm{dn}}}\right\}\right\}$ as follows:

(20) $$\begin{array}{l} \theta_{l}∼p\left(\theta_{l}|w,z^{\text{o}},z^{\text{c}},z^{\text{p}},z^{\text{a}},F,A,\gamma \right)\\ \;\propto \overset{D}{\underset{d=1}{\prod }}\;\overset{N_{d}}{\underset{n=1}{\prod }}\text{ Cat}\;\left(w_{\mathrm{dn}}|\theta_{l=\left(F_{\mathrm{dn}},z_{dA_{d}}^{F_{\mathrm{dn}}}\right)}\right)\;\text{Dir}\left(\theta_{l}|\gamma \right)\;\\ \;\propto \text{Dir}\left(\theta_{l}|w_{l},\gamma \right) \end{array}$$

where *w* denotes the set of all the words, *F* denotes the set of frames of all the sentences, and *w_l_* denotes the set of all the words of the word category $l=\left(F_{\mathrm{dn}},z_{dA_{d}}^{F_{\mathrm{dn}}}\right)$ in *n* ∈ {1, 2, … , *N_d_*} and *d* ∈ {1, 2, … , *D*}. A latent variable $z_{\mathrm{dm}}^{\text{o}}$ of the object category is sampled for each *m* ∈ {1, 2, … , *M_d_*} and *d* ∈ {1, 2, … , *D*} as follows:

(21) $$\begin{array}{l} z_{\mathrm{dm}}^{\text{o}}∼p\left(z_{\mathrm{dm}}^{\text{o}}|w_{d},z_{d}^{\text{c}},z_{d}^{\text{p}},z_{d}^{\text{a}},z_{-\mathrm{dm}}^{\text{o}},\theta ,F_{d},A_{d},o_{\mathrm{dm}},\phi^{\text{o}},\pi^{\text{o}}\right)\\ \;\propto \overset{N_{d}}{\underset{n=1}{\prod }}\text{ Cat }\left(w_{\mathrm{dn}}|\theta_{l=\left(F_{\mathrm{dn}},z_{dA_{d}}^{F_{\mathrm{dn}}}\right)}\right)\text{ Gauss }\left(o_{\mathrm{dm}}|\phi_{z_{\mathrm{dm}}^{\text{o}}}^{\text{o}}\right)\;\text{Cat}\;\left(z_{\mathrm{dm}}^{\text{o}}|\pi^{\text{o}}\right), \end{array}$$

where *w_d_* is a sequence of words in the *d*-th trial and $z_{-\mathrm{dm}}^{\text{o}}$ is the set of indicates of the object categories without $z_{\mathrm{dm}}^{\text{o}}$ in the *d*-th trial. A latent variable $z_{\mathrm{dm}}^{\text{c}}$ of the color category is sampled for each *m* ∈ {1, 2, … , *M_d_*} and *d* ∈ {1, 2, … , *D*} as follows:

(22) $$\begin{array}{l} z_{\mathrm{dm}}^{\text{c}}∼p\left(z_{\mathrm{dm}}^{\text{c}}|w_{d},z_{d}^{\text{o}},z_{d}^{\text{p}},z_{d}^{\text{a}},z_{-\mathrm{dm}}^{\text{c}},\theta ,F_{d},A_{d},c_{\mathrm{dm}},\phi^{\text{c}},\pi^{\text{c}}\right)\\ \;\propto \overset{N_{d}}{\underset{n=1}{\prod }}\text{ Cat }\left(w_{\mathrm{dn}}|\theta_{l=\left(F_{\mathrm{dn}},z_{dA_{d}}^{F_{\mathrm{dn}}}\right)}\right)\text{ Gauss }\left(c_{\mathrm{dm}}|\phi_{z_{\mathrm{dm}}^{\text{c}}}^{\text{c}}\right)\text{ Cat }\left(z_{\mathrm{dm}}^{\text{c}}|\pi^{\text{c}}\right), \end{array}$$

where $z_{-\mathrm{dm}}^{\text{c}}$ is the set of indicates of the object categories without $z_{\mathrm{dm}}^{\text{c}}$ in the *d*-th trial. A latent variable $z_{\mathrm{dm}}^{\text{p}}$ of the position category is sampled for each *m* ∈ {1, 2, … , *M_d_*} and *d* ∈ {1, 2, … , *D*} as follows:

(23) $$\begin{array}{l} z_{\mathrm{dm}}^{\text{p}}∼p\left(z_{\mathrm{dm}}^{\text{p}}|w_{d},z_{d}^{\text{o}},z_{d}^{\text{c}},z_{d}^{\text{a}},z_{-\mathrm{dm}}^{\text{p}},\theta ,F_{d},A_{d},p_{\mathrm{dm}},\phi^{\text{p}},\pi^{\text{p}}\right)\\ \;\propto \overset{N_{d}}{\underset{n=1}{\prod }}\text{ Cat }\left(w_{\mathrm{dn}}|\theta_{l=\left(F_{\mathrm{dn}},z_{dA_{d}}^{F_{\mathrm{dn}}}\right)}\right)\text{ Gauss }\left(p_{\mathrm{dm}}|\phi_{z_{\mathrm{dm}}^{\text{p}}}^{\text{p}}\right)\text{ Cat }\left(z_{\mathrm{dm}}^{\text{p}}|\pi^{\text{p}}\right), \end{array}$$

where $z_{-\mathrm{dm}}^{\text{p}}$ is the set of indicates of the object categories without $z_{\mathrm{dm}}^{\text{p}}$ in the *d*-th trial. A latent variable $z_{d}^{\text{a}}$ of the action category is sampled for each *d* ∈ {1, 2, … , *D*} as follows:

(24) $$\begin{array}{l} z_{d}^{\text{a}}∼p\left(z_{d}^{\text{a}}|w_{d},z_{d}^{\text{o}},z_{d}^{\text{c}},z_{d}^{\text{p}},\theta ,F_{d},A_{d},a_{d},p_{d},\phi^{\text{a}},\pi^{\text{a}}\right)\\ \;\propto \overset{N_{d}}{\underset{n=1}{\prod }}\text{ Cat }\left(w_{\mathrm{dn}}|\theta_{l=\left(F_{\mathrm{dn}},z_{dA_{d}}^{F_{\mathrm{dn}}}\right)}\right)\text{ Gauss }\left(a_{d}|\phi_{z_{d}^{\text{a}}}^{{\text{a}}^{\prime }}\right)\text{ Cat }\left(z_{d}^{\text{a}}|\pi^{\text{a}}\right), \end{array}$$

where *p_d_* is the set of position data in the *d*-th trial. A latent variable *F_d_* representing the sensory-channels of words in a sentence is sampled for each *d* ∈ {1, 2, … , *D*} as follows:

(25) $$\begin{array}{l} F_{d}∼p\left(F_{d}|w,z^{\text{o}},z^{\text{c}},z^{\text{p}},z^{\text{a}},\theta ,A,\lambda \right)\\ \;\propto \overset{N_{d}}{\underset{n=1}{\prod }}\text{ Cat }\left(w_{\mathrm{dn}}|\theta_{l=\left(F_{\mathrm{dn}},z_{dA_{d}}^{F_{\mathrm{dn}}}\right)}\right)\text{ Unif }\left(F_{d}|\lambda \right). \end{array}$$

### 3.4 Action Generation and Attention Selection

In this section, we describe the approach that selects an action and an object of attention from the human spoken sentence. A robot capable of learning word meanings accurately is considered to be able to understand human instruction more accurately. In an action generation task, the robot performs an action *a_d_* based on word meanings and multiple categories Θ from observed information *w_d_*, *o_d_*, *c_d_*, and *p_d_*. In this case, the robot can use the set of model parameters Θ learned by using Gibbs sampling in the CSL task. In the action generation task, we maximize the following equation:

(26) $$\begin{array}{l} \underset{a_{d}}{\text{argmax}}\;p\left(a_{d}|w_{d},o_{d},c_{d},p_{d},\theta ,\left\{\phi \right\},\left\{\pi \right\},\lambda \right)\\ \;=\underset{a_{d}}{\text{argmax}}\sum_{A_{d}}\sum_{z_{d}^{\text{a}}}\;p\left(a_{d}|\phi^{\text{a}},z_{d}^{\text{a}},p_{d},A_{d}\right)\\ \;\times \;p\left(A_{d},z_{d}^{\text{a}}|w_{d},o_{d},c_{d},p_{d},\theta ,\left\{\phi \right\},\left\{\pi \right\},\lambda \right). \end{array}$$

In practice, this maximization problem is separated into two approximation processes, because it is difficult to maximize equation (26) directly.

(1) The first process is the maximization of the attention *A_d_* and the index of the action category $z_{d}^{\text{a}}$
  (27) $$A_{d}^{*},{z_{d}^{\text{a}}}^{*}=\underset{A_{d},z_{d}^{\text{a}}}{\text{argmax}}\;p\left(A_{d},z_{d}^{\text{a}}|w_{d},o_{d},c_{d},p_{d},\theta ,\left\{\phi \right\},\left\{\pi \right\},\lambda \right).$$
  The probability distribution of equation (27) is represented by the following equation:
  (28) $$\begin{array}{l} p\left(A_{d},z_{d}^{\text{a}}|w_{d},o_{d},c_{d},p_{d},\theta ,\left\{\phi \right\},\left\{\pi \right\},\lambda \right)\\ \;\propto p\left(A_{d}=m\right)p\left(z_{d}^{\text{a}}|\pi^{\text{a}}\right)\underset{M_{d}}{\prod }\;\underset{z_{\mathrm{dm}}^{\text{o}}}{\sum }\;\underset{z_{\mathrm{dm}}^{\text{c}}}{\sum }\;\underset{z_{\mathrm{dm}}^{\text{p}}}{\sum }\\ \text{Gauss}\;\left(o_{\mathrm{dm}}|\phi_{z_{\mathrm{dm}}^{\text{o}}}^{\text{o}}\right)\;\text{Cat}\;\left(z_{\mathrm{dm}}^{\text{o}}|\pi^{\text{o}}\right)\\ \text{Gauss}\;\left(c_{\mathrm{dm}}|\phi_{z_{\mathrm{dm}}^{\text{c}}}^{\text{c}}\right)\;\text{Cat}\;\left(z_{\mathrm{dm}}^{\text{c}}|\pi^{\text{c}}\right)\\ \text{Gauss}\;\left(p_{\mathrm{dm}}|\phi_{z_{\mathrm{dm}}^{\text{p}}}^{\text{p}}\right)\;\text{Cat}\;\left(z_{\mathrm{dm}}^{\text{p}}|\pi^{\text{p}}\right)\;\\ \;\left[\sum_{F_{d}}\text{ Unif}\left(F_{d}|\lambda \right)\prod_{N_{d}}\;\text{Cat}\;\left(w_{\mathrm{dn}}|\theta_{l=\left(F_{\mathrm{dn}},z_{dA_{d}}^{F_{\mathrm{dn}}}\right)}\right)\right]. \end{array}$$
  Then, we assumed *p*(*A_d_* = *m*) = 1/*M_d_* as equal probability for the number of objects.
(2) The second process is the maximization of the action *a_d_* using $A_{d}^{*}$ and ${z_{d}^{\text{a}}}^{*}$
  (29) $$\begin{array}{l} a_{d}^{*}=\underset{a_{d}}{\text{argmax}}\;p\left(a_{d}|\phi^{\text{a}},{z_{d}^{\text{a}}}^{*},p_{d},A_{d}^{*}\right)\\ \;=\underset{a_{d}}{\text{argmax}}\text{ Gauss}\;\left(a_{d}|\phi_{{z_{d}^{\text{a}}}^{*}}^{{\text{a}}^{\prime }}\right)\\ \;=\mu_{{z_{d}^{\text{a}}}^{*}}^{{\text{a}}^{\prime }}, \end{array}$$
  where the mean vector of the Gaussian distribution of the action category ${z_{d}^{\text{a}}}^{*}$ is denoted as $\mu_{{z_{d}^{\text{a}}}^{*}}^{{\text{a}}^{\prime }}$.

### 3.5 Description of the Current Situation and Self-Action by the Robot

In this section, we describe the approach followed by the description task representing the current situation and the self-action of the robot. We consider a robot capable of learning word meanings accurately to be able to describe the current situation and self-action more accurately. In the action description task, the robot utters a sentence *w_d_* regarding a self-action *a_d_* and observed information *o*_d_, *c_d_*, and *p_d_* based on word meanings and multiple categories Θ. In this case, the robot can use the set of model parameters Θ learned by using Gibbs sampling in the CSL task. In the action description task, we maximize the following equation:

(30) $$\begin{array}{l} \underset{w_{d}}{\text{argmax}}\;p\left(w_{d}|a_{d},o_{d},c_{d},p_{d},\theta ,\left\{\phi \right\},\left\{\pi \right\},F_{d},A_{d}\right)\\ \;\propto \text{argma}{\text{x}}_{w_{d}}\underset{z_{d}^{\text{a}}}{\sum }\;\underset{z_{dA_{d}}^{\text{o}}}{\sum }\;\underset{z_{dA_{d}}^{\text{c}}}{\sum }\;\underset{z_{dA_{d}}^{\text{p}}}{\sum }\\ \text{Gauss}\;\left(a_{d}|\phi_{z_{d}^{\text{a}}}^{{\text{a}}^{\prime }}\right)\;\text{Cat}\;\left(z_{d}^{\text{a}}|\pi^{\text{a}}\right),\\ \text{Gauss}\;\left(o_{dA_{d}}|\phi_{z_{dA_{d}}^{\text{o}}}^{\text{o}}\right)\;\text{Cat}\;\left(z_{dA_{d}}^{\text{o}}|\pi^{\text{o}}\right)\\ \text{Gauss}\;\left(c_{dA_{d}}|\phi_{z_{dA_{d}}^{\text{c}}}^{\text{c}}\right)\;\text{Cat}\;\left(z_{dA_{d}}^{\text{c}}|\pi^{\text{c}}\right)\\ \text{Gauss}\;\left(p_{dA_{d}}|\phi_{z_{dA_{d}}^{\text{p}}}^{\text{p}}\right)\;\text{Cat}\;\left(z_{dA_{d}}^{\text{p}}|\pi^{\text{p}}\right)\\ \;\underset{N_{d}}{\prod }\text{ Cat}\;\left(w_{\mathrm{dn}}|\theta_{l=\left(F_{\mathrm{dn}},z_{dA_{d}}^{F_{\mathrm{dn}}}\right)}\right). \end{array}$$

If the frame of the sentence is decided, e.g., *F_d_* = (a, p, c, o), equation (30) is represented as the following:

(31) $$\text{Equation }\left(30\right)=\underset{N_{d}}{\prod }\;\underset{w_{\mathrm{dn}}}{\text{argmax}}\underset{z_{dA_{d}}^{F_{\mathrm{dn}}}}{\sum }\text{ Gauss}\;\left(x_{dA_{d}}^{F_{\mathrm{dn}}}|\phi_{z_{dA_{d}}^{F_{\mathrm{dn}}}}^{F_{\mathrm{dn}}}\right)\times \;\text{Cat}\;\left(z_{dA_{d}}^{F_{\mathrm{dn}}}|\pi^{F_{\mathrm{dn}}}\right)\;\text{Cat}\;\left(w_{\mathrm{dn}}|\theta_{l=\left(F_{\mathrm{dn}},z_{dA_{d}}^{F_{\mathrm{dn}}}\right)}\right),$$

where $x_{dA_{d}}^{F_{\mathrm{dn}}}$ denotes data of the sensory-channel *F_dn_* in the object number *A_d_*, i,e., *a_d_*, $p_{dA_{d}}$, $c_{dA_{d}}$, or $o_{dA_{d}}$. Therefore, equation (30) can be divided into the equations of finding a maximum value for each word.

## 4 Experiment I: Simulation Environment

We performed the experiments described in this section using the iCub simulator (Tikhanoff et al., 2008). In Section 4.1, we describe the difference in the conditions of the methods that are used for comparison purposes. In Section 4.2, we describe the CSL experiment. In Section 4.3, we describe the experiment involving the action generation task. In Section 4.4, we describe the experiment relating to the action description task.

### 4.1 Comparison Methods

We evaluated our proposed method by comparing its performance with that of two other methods.

(A) The proposed method.
  This method has a mutual exclusivity constraint between the word and the sensory-channel (MEC-I and II), determining that each sensory-channel occurs only once in each sentence. For example, if the number of words in a sentence is *N_d_* = 4, *F_d_* can become a sequence such as (a, c, p, o), (a, p, c, o), or (p, c, o, a). Possible values of *F_d_* are constrained by λ as a permutation of four sensory-channels. The number of permutations is ${}_{4}{\text{P}}_{N_{d}}=4!∕\left(4-N_{d}\right)!$.
(B) The proposed method without the mutual exclusivity constraint (w/o MEC-II).
  This method does not have the mutual exclusivity constraint (MEC-II). This means that several words in a sentence may relate to the same sensory-channel. For example, if the number of words in a sentence is *N_d_* = 4, *F_d_* can become a sequence such as (a, o, c, o), (a, p, p, o), or (o, o, o, o) in addition to the above example of (A). Possible values of *F_d_* are constrained by λ as a repeated permutation of four sensory-channels. The number of repeated permutations is ${}_{4}\Pi_{N_{d}}=4^{N_{d}}$. In this case, the robot needs to consider additional pairs of relationships between the sensory-channel and word compared to method (A).
(C) The multilayered multimodal latent Dirichlet allocation (mMLDA) (Attamimi et al., 2016).
  This method is based on mMLDA. In this research, this method was modified from the actual mMLDA to apply to our task and the proposed method. In particular, the emission probability for each sensory-channel is changed from a categorical distribution to a Gaussian distribution. This means the multimodal categorization methods are based on a Gaussian distribution for each sensory-channel and a categorical distribution for word information. This method relates all observed words in a situation to all observed sensory-channel information in the situation. This method neither has the mutual exclusivity constraint (MEC-I and II) nor does it select the sensory-channel by words, i.e., *F_d_* is not estimated.

### 4.2 Cross-Situational Learning

#### 4.2.1 Experimental Procedure and Conditions

**Figure 3:**
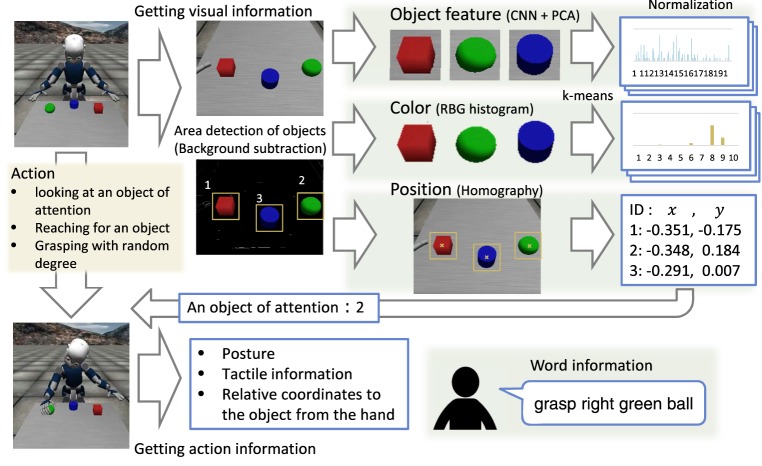
Procedure for obtaining and processing data.

We conducted an experiment to learn the categories for each sensory-channel and the words associated with each category. Figure 3 shows the procedure for obtaining and processing data. We describe the experimental procedure for CSL as follows:

1. The robot takes the initial position and posture. Some objects are placed on the table.
2. The robot acquires a visual image of the table. Subsequently, the robot detects object areas by using background subtraction. The detected object areas are cut out as object images of 64 × 64 pixels. The robot obtains the number of objects on the table.
3. The robot extracts object features, color features, and object positions. We used the deep learning framework Caffe (Jia et al., 2014) for convolutional neural networks (CNNs) (Krizhevsky et al., 2012) as an object feature extractor. We used a pre-trained CNN, i.e., CaffeNet trained by using ImageNet Large Scale Visual Recognition Challenge 2012 as the dataset. The object features are obtained from the fully connected FC6 layer (4096-dimensions) in CaffeNet. After that, the object features are reduced by principal component analysis (PCA). In terms of color features, the RGB histogram is vector quantized by k-means and normalized. The position data are converted into the world coordinate by homography. The position data are two-dimensional to represent the plane of the table.
4. The robot performs an action including a little randomness to an object of attention. The difference and uncertainty in the robot’s action are represented by this randomness. First, the robot moves its eye-gaze to an object of attention. The object is selected randomly. Next, the robot moves its right hand to the coordinates of the target object by using inverse kinematics. A little random noise is added to a target position of the end-effector of the right hand. In many cases, the robot moves its right hand after looking at the object. The robot rarely refrains from moving its hands, looks at the object, which means the action of “look-at.” When the hand approaches the position of the target object, the robot bends its fingers. The rate at which it bends its fingers is selected randomly. The five fingers move in synchronization. After the action is completed, the robot acquires the data relating to this action, including data relating to the posture, tactile data, and the relative coordinates of the object from its right hand. The action data are 38-dimensional and include the position of the right hand relative to the object (3-dim.), the rate at which the finger bends (1-dim.), the joint angles of the head, right_arm, and torso (6, 16, 3-dim.), and tactile information of the right hand (9-dim.). The action data is normalized to [0,1] for each dimension.
5. When the robot completes an action, the human tutor speaks a sentence about the object of attention and the action of the robot. The sentence contains the word related to each sensory-channel once, e.g., “touch left red box.” In this task, the number of words in the sentence is indicated by a number ranging from zero to four. Zero means that the tutor did not speak a sentence.

The above process is carried out many times in different situations. The robot learns multiple categories and word meanings by using multimodal data observed in many trials.

The number of trials was *D* = 20 and 40 for CSL. The number of objects *M_d_* on the table for each trial was a number from one to three. The number of words *N_d_* in the sentence was a number from zero to four. We assume that a word related to each sensory-channel is spoken only once in each sentence. The word order in the sentences was changed. This experiment used 14 kinds of words: “reach,” “touch,” “grasp,” “look-at,” “front,” “left,” “right,” “far,” “green,” “red,” “blue,” “box,” “cup,” and “ball.” The upper limit number of the categories for each sensory-channel was K = 10, i.e., the number of word distributions was *L* = 40. The number of iterative cycles used for Gibbs sampling was 200. The hyperparameters were α = 1.0, γ = 0.1, $m_{0}=O_{x_{\dim}}$, κ_0_ = 0.001, *V*_0_ = diag(0.01, 0.01), and *v*0 = *x_dim_* + 2, where the number of dimensions for each sensory-channel *x* is denoted as *x_dim_* and the zero vector in *x_dim_* dimensions is denoted as $O_{x_{\dim}}$. PCA is used to reduce the object features to 30 dimensions. The color features are quantized to 10 dimensions by k-means.

We describe the criteria of words uttered for action category as follows: “reach” corresponds to the robot extending its right hand toward an object and the robot’s finger does not make contact with an object; “touch” corresponds to the robot touching an object and its finger is relatively opened; “grasp” corresponds to the robot’s hand holding firmly an object; “look-at” corresponds to the robot not moving its right hand and it focuses on an object of attention only. Based on these criteria, the tutor determines an action word. In particular, “reach” and “touch” are similar; the only difference is whether the hand touches the object or not.

We evaluate the estimation accuracy of the learning results by using uncertain teaching sentences. Each sentence contains four words or fewer in different order. We compare the accuracy of three methods by reducing the word information. In addition, the number of learning trials is changed. We compared the accuracy by changing the number of trials. We evaluated the methods according to the following metrics.

- Adjusted Rand index (ARI)
  We compare the matching rate between the estimated latent variables *z* for each sensory-channel and the true categorization results. The evaluation of this experiment uses the ARI (Hubert and Arabie, 1985), which is a measure of the degree of similarity between two clustering results.
- Estimation accuracy rate of *F_d_* (EAR)
  The evaluation of the estimation results of the sensory-channels corresponding to the words are determined as follows:
  (32) $$\text{EAR}=1-\frac{\text{The number of estimation errors}}{\text{The number of all of uttered words}}.$$

#### 4.2.2 Learning Results and Evaluation

**Figure 4:**
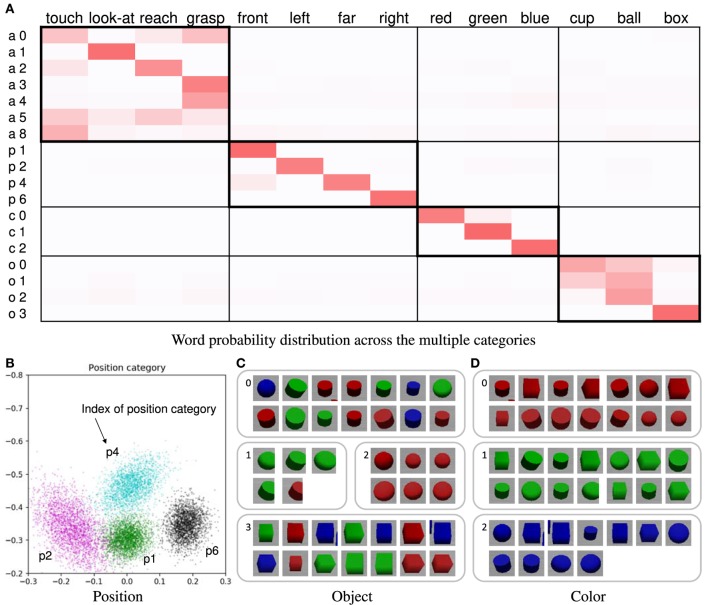
**(A)** Word probability distribution across the multiple categories; darker shades represent higher probability values. The pair consisting of a letter and a number on the left of the table is the index of the word distribution, which represents the sensory-channel related to the word distribution and the index of the category. Note that category indices are not shown; they are merged and not used because the number of the categories is automatically estimated by the nonparametric Bayesian method. **(B)** Learning result of the position category; for example, the index of position category p1 corresponds to the word “front.” The point group of each color represents each Gaussian distribution of the position category. The crosses in the different colors represent the object positions of the learning data. Each color represents a position category. **(C)** Example of categorization results of object category; **(D)** example of categorization results of color category.

The learning results obtained by using the proposed method are presented here. Forty trials were used. In this case, the number of words was four in all utterance sentences. Figure 4A shows the word probability distributions θ. Higher probability values are represented by darker shades. If the relationship between the word and sensory-channel can be estimated correctly, the ranges within thick-bordered boxes show higher probabilities. For example, the action categories show higher probabilities for words of action (“touch,” “look-at,” “reach,” and “grasp”). The categories of the other sensory-channels are also the same. In the position and color categories, the estimated number of categories was equal to the number of types of words representing the sensory-channel. In the action category, the words “touch,” “reach,” and “grasp” were associated across several categories. In addition, these words were confused with each other. We considered actions representing these words to be ambiguous and similar. On the other hand, we considered the reason why these actions were divided into several categories to be a change in posture information depending on the position of the target object. Figure 4B shows the learning result for the position category *ϕ^p^*. For example, the position category p1 is associated with the word “front” (see Figures 4A,B). Figures 4C,D show examples of the categorization results for the object and color categories. The object categorization result was not perfect. We considered the robot to find it difficult to clearly distinguish objects of different shapes because the 3D-models of the objects had simple shapes. The color categorization result was perfect. In this case, *F_d_* was correctly estimated in all of the trials. The results demonstrate that the proposed method was able to accurately associate each word with its respective sensory-channel.

We performed the learning scenarios 10 times for each method. Tables 1A,B show the evaluation values of the experimental results for 20 and 40 trials. The rate of omitted words (ROW), which is expressed as a percentage, represents the uncertainty of teaching sentences. For example, the total number of words is 80 when ROW is 0%, 64 words for 20%, 48 words for 40%, and 32 words for 60% in 20 trials. Also, the total number of words is 160 for a ROW value of 0% and 96 words for 40% in 40 trials. ARI_a, ARI_p, ARI_o, and ARI_c are the ARI values of the action, position, object, and color category, respectively. The EAR values of mMLDA were not calculated because this method does not have *F_d_*. If the ROW value is 100 (no word), the three methods will be equivalent as ALL, i.e., the GMM for each sensory-channel. We described the ARI values of ALL as reference values because ALL is not CSL. The EAR value obtained for the proposed method was higher than that obtained for the other methods. When the ROW decreased, i.e., the word information increased, the evaluation values tended to increase. Particularly, the result for the position category was favorably affected by the increase in word information for categorization. In addition, when the number of trials increased, the evaluation values tended to increase. This result suggests that the robot is able to learn the word meanings more effectively by accumulating more experience even in more complicated and uncertain situations. When the number of words was small (i.e., the ROW value is 40 or 60%), the difference between the EAR values of methods (A) and (B) was small (approximately 0.02) in 20 trials. However, when the number of words was large, the difference between the EAR values of methods (A) and (B) increased, and the EAR value of the method (A) was larger than that of (B). As a result, when the number of words was small, e.g., sentences including one or two words, there was almost no influence of the presence or absence of the MEC-II because the number of possible values of *F_d_* of the methods (A) and (B) were close. On the other hand, when the number of words was large, e.g., sentences including four words, the MEC-II worked well because the number of possible values of *F_d_* of the method (A) was narrowed properly down.

**Table 1:** Experimental results of the CSL task for 20 and 40 trials.

Method	ROW	ARI_a	ARI_p	ARI_o	ARI_c	EAR_*F_d_*
**(A) 20 trials**						
Proposed	0	0.300	0.606	0.408	0.782	**0.970**
w/o MEC-II	0	0.317	0.648	0.338	0.805	**0.759**
mMLDA	0	0.316	0.428	0.277	0.756	–
Proposed	20	0.290	0.564	0.332	0.762	0.727
w/o MEC-II	20	0.342	0.486	0.436	0.755	0.598
mMLDA	20	0.267	0.494	0.369	0.776	–
Proposed	40	0.324	0.493	0.354	0.780	0.556
w/o MEC-II	40	0.318	0.486	0.347	0.812	0.529
mMLDA	40	0.356	0.479	0.312	0.771	–
Proposed	60	0.282	0.460	0.295	0.783	0.381
w/o MEC-II	60	0.311	0.454	0.326	0.750	0.406
mMLDA	60	0.294	0.487	0.403	0.724	–
ALL	100 (no word)	0.325	0.431	0.346	0.751	–
**(B) 40 trials**						
Proposed	0	0.375	0.540	0.366	0.870	**0.989**
w/o MEC-II	0	0.383	0.524	0.333	0.805	0.834
mMLDA	0	0.388	0.594	0.377	0.822	–
Proposed	40	0.368	0.543	0.313	0.835	**0.867**
w/o MEC-II	40	0.417	0.577	0.320	0.842	0.780
mMLDA	40	0.340	0.600	0.377	0.856	–

*Bold and underscore indicate the highest evaluation values, and bold indicates the second highest evaluation values*.

### 4.3 Action Generation Task

#### 4.3.1 Experimental Procedure and Conditions

In this experiment, the robot generates the action regarding the sentence spoken by the human tutor. The robot uses the learning results of the CSL task in Section 4.2. The robot selects the object of attention from among the objects on the table. In addition, the robot performs the action on the object of attention. In this task, the robot cannot use joint attention. Therefore, the robot needs to overcome both the problems of CSL-I and II. We describe the process of action generation as follows:

1. The robot takes the initial position and posture. Some objects are placed on the table.
2. The tutor speaks a sentence about an action that should be performed by the robot. The robot recognizes the tutor’s spoken sentence.
3. The robot detects the objects on the table. The robot obtains object, color features, and position data by the same process as in Section 4.2.1 (step 3).
4. The robot selects the action category and the object of the attention by using equation (26). The robot calculates the target position by using equation (29).
5. The robot directs its eye-gaze to the object of attention, and the robot performs an action on the object of attention.

The above process is carried out many times on different sentences.

We compare the three methods by quantitative evaluation on the action generation task. We evaluate the accuracy of the selection of the object of attention. In addition, we evaluate the accuracy of an action of the robot based on questionnaire evaluation by participants. The robot generates an action from the tutor’s spoken sentence in a situation. Participants check videos of the action generated by the robot and select a word representing the robot’s action. We calculate the word accuracy rate (WAR) of the words selected by participants and the true words spoken by the tutor. In addition, we calculate the object accuracy rate (OAR) representing the rate at which the robot correctly selected the object instructed by the tutor.

We performed action generation tasks for a total of 12 different test-sentences. The test-sentences included four words representing the four sensory-channels. This placement of objects on the table was not used during the learning trials. In addition, the word order of sentences uttered during the action generation task is different from the word order of sentences uttered during the CSL task. The eight participants checked 36 videos of the robot’s actions.

#### 4.3.2 Results

**Figure 5:**
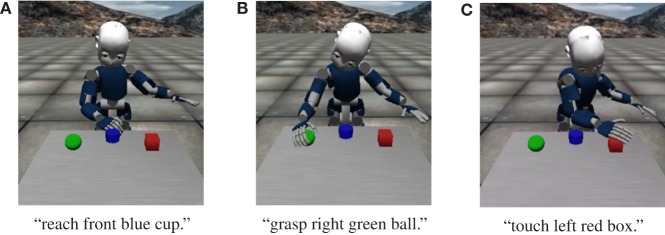
Example of results of the action generation task in the iCub simulator. **(A)** Reach front blue cup. **(B)** Grasp right green ball. **(C)** Touch left red box.

Figure 5 shows three examples of the action generation results of the proposed method. Figure 5A shows the result of action generation by the robot in response to the sentence “reach front blue cup.” Figure 5B shows the result of action generation by the robot in response to the sentence “grasp right green ball.” Figure 5C shows the result of action generation by the robot in response to the sentence “touch left red box.” Table 2 shows the results of the quantitative evaluation of the action generation task. The proposed method enabled the robot to accurately select the object. As a result of the proposed method, the object indicated and the object selected by the robot coincided in all sentences. In addition, the proposed method showed the highest values for both WAR and OAR. Therefore, the robot could select an appropriate object and could perform an action even in situations and for sentences not used for CSL.

**Table 2:** Results of evaluation values for the action generation using the results of the CSL for 40 trials (ROW is 0%).

Method	WAR	OAR
Proposed	**0.604**	**1.000**
w/o MEC-II	**0.510**	**0.917**
mMLDA	0.260	0.667

*Bold and underscore indicate the highest evaluation values, and bold indicates the second highest evaluation values*.

### 4.4 Action Description Task

#### 4.4.1 Experimental Procedure and Conditions

In HRI, the ability of the robot to use the acquired word meanings for a description of the current situation is important. In this experiment, the robot performs an action and speaks the sentence corresponding to this action. In other words, the robot explains self-action by using a sentence. The robot uses the learning results of the CSL task in Section 4.2. We describe the process of action description as follows:

1. The robot takes the initial position and posture. Some objects are placed on the table.
2. The robot detects the objects on the table. The robot obtains the object, color features, and position data by the same process as in Section 4.2.1 (step 3).
3. The robot selects the object of attention randomly. The robot directs its eye-gaze to the object of attention, and the robot performs an action on the object of attention by using the same process as in Section 4.2.1 (step 4).
4. When the robot completes an action, it utters a sentence about this action.

The above process is carried out many times on different actions. We performed action description tasks for a total of 12 actions. This placement of objects on the table was not used during the learning trials. The robot generates a sentence consisting of four words that include the four sensory-channels. The word order in the sentence is fixed as *F_d_* = (a, p, c, o).

We compare the three methods by quantitative evaluation of the action description task. We evaluate the F1-measure and the accuracy (ACC) between the sentence generated by the robot and the correct sentence decided by the tutor. The evaluation values are calculated by generating the confusion matrix between the predicted words and true words.

#### 4.4.2 Results

**Figure 6:**
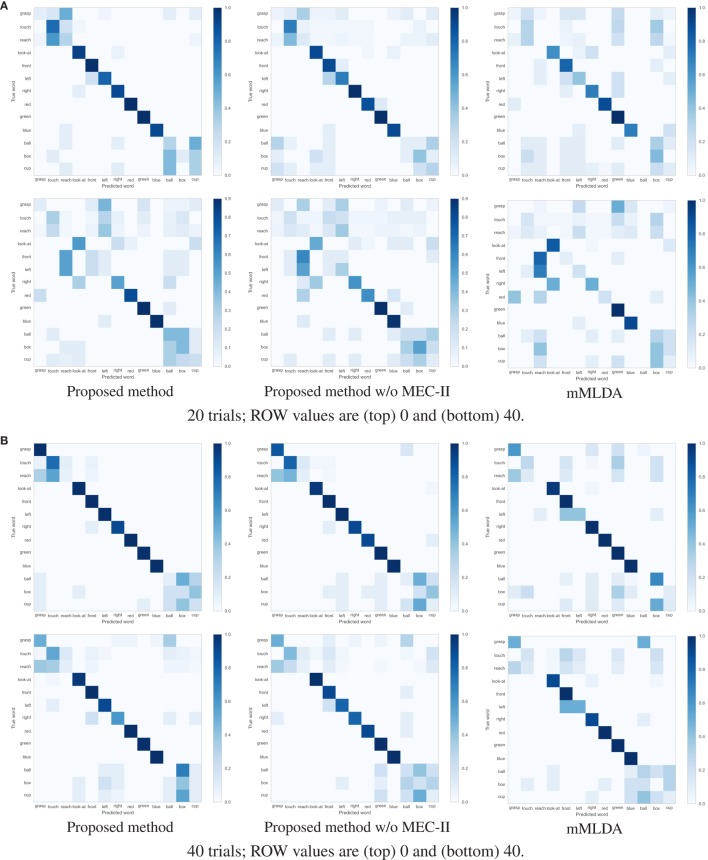
Confusion matrix of results of the action description task using the learning result for 20 and 40 trials. **(A)** 20 trials; ROW values are (top) 0 and (bottom) 40. **(B)** 40 trials; ROW values are (top) 0 and (bottom) 40.

Table 3 show the F1-measure and ACC values of the action description task using the learning results under the different conditions. The proposed method showed the highest evaluation values. Figures 6A,B shows the confusion matrices of the results of the action description task using the learning result for 20 and 40 training trials. Overall, the robot confused the words “reach” and “touch” similar to the learning result in Figure 4A. The robot had difficulties in distinguishing between “reach” and “touch.” In other words, this result suggests that these words were learned as synonyms. When the ROW increased, the evaluation values decreased. For the ROW value of 40% obtained for 20 trials, the robot confused words related to the action and position categories. This could be explained by considering that the robot misunderstood the correspondence between the word and the sensory-channel because the word information was insufficient and uncertain during CSL with the ROW value of 40% and 20 trials. On the other hand, an increase in the number of learning trials resulted in an increase in the evaluation values. Even if the robot is exposed to uncertain utterances, the robot can explain self-action more accurately by gaining more experience. As a result, the robot could acquire the ability to explain self-action by CSL based on the proposed method.

**Table 3:** Experimental results of action description task for 20 and 40 trials.

Method	Trials	ROW	F1	ACC
Proposed	20	0	**0.586**	**0.660**
w/o MEC-II	20	0	**0.534**	**0.613**
mMLDA	20	0	0.401	0.469
Proposed	20	40	**0.388**	**0.425**
w/o MEC-II	20	40	**0.343**	**0.369**
mMLDA	20	40	0.319	0.352
Proposed	40	0	**0.663**	**0.692**
w/o MEC-II	40	0	**0.642**	**0.671**
mMLDA	40	0	0.474	0.560
Proposed	40	40	**0.588**	**0.623**
w/o MEC-II	40	40	**0.548**	**0.606**
mMLDA	40	40	0.479	0.569

*Bold and underscore indicate the highest evaluation values, and bold indicates the second highest evaluation values*.

## 5 Experiment II: Real iCub Environment

In this section, we describe the experiment that was conducted by using the real iCub robot. The real-world environment involves more complexity than the simulation environment. We demonstrate that results similar to those of the simulator experiment can be obtained even in a more complicated real environment. We compare three methods, as in Section 4.1. In Section 5.1, we describe the experiment to assess cross-situational learning. In Section 5.2, we describe the experiment relating to the action generation task. In Section 5.3, we describe the experiment relating to the action description task.

### 5.1 Cross-Situational Learning

#### 5.1.1 Conditions

**Figure 7:**
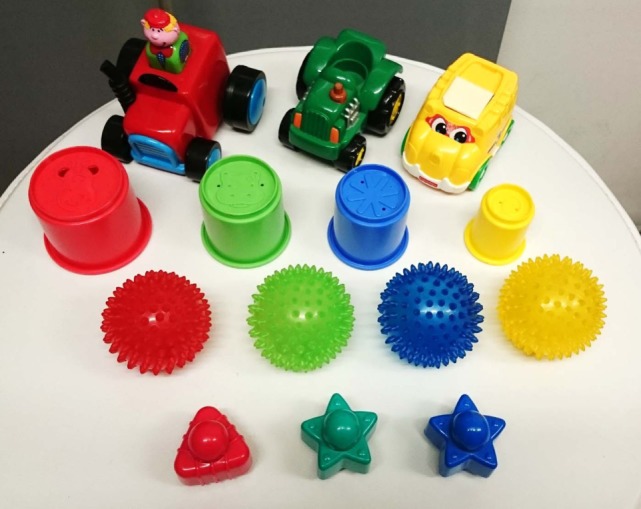
All of the objects used in the real experiments (14 objects including four types and four colors).

The experimental procedure is the same as in Section 4.2.1. We use ARI and EAR as evaluation values. Figure 7 shows all of the objects that were used in the real environment. We used 14 different objects including four types (car, cup, ball, and star) and four colors (red, green, blue, and yellow). In the simulation environment, the same type objects had the same shapes. In the real environment, objects of the same type include different shapes. In particular, all the car objects have different shapes, the cup objects have different sizes, and the star objects include one different shape. This experiment used 16 kinds of words: “reach,” “touch,” “grasp,” “look-at,” “front,” “left,” “right,” “far,” “green,” “red,” “blue,” “yellow,” “car,” “cup,” “ball,” and “star.” The number of trials was *D* = 25 and 40 for CSL. The number of objects *M_d_* on the table for each trial was a number ranging from one to three. The number of words *N_d_* in the sentence was a number ranging from zero to four. We assume that a word related to each sensory-channel is spoken only once in each sentence. The word order in the sentences was changed. Object features are reduced to 65 dimensions by PCA. Color features are quantized to 10 dimensions by k-means. The upper limit number of the categories for each sensory-channel was *K* = 10, i.e., the upper limit for the number of word distributions was *L* = 40. The hyperparameters were α = 1.0, γ = 0.1, $m_{0}=O_{x_{\dim}}$, κ_0_ = 0.001, *V*_0_ = diag(0.01, 0.01), and *v*_0_ = *x_dim_* + 2. The number of iterative cycles used for Gibbs sampling was 200.

#### 5.1.2 Learning Results and Evaluation

**Figure 8:**
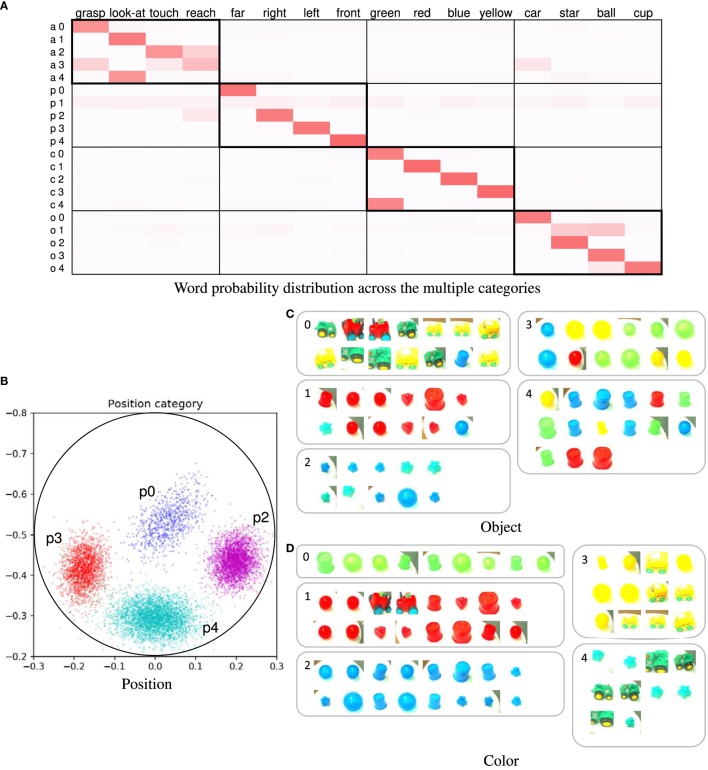
**(A)** Word probability distribution across the multiple categories; **(B)** learning result of position category; each color of the point group represents each of the Gaussian distributions of the position category. The crosses of each color represent the object positions of the learning data. Each color represents a position category. The circle represents the area of the white circular table. **(C)** Example of categorization results of object category; **(D)** example of categorization results of color category.

The example we describe is the learning result of 25 trials and for a ROW value of 9%. In this case, the number of categories was set to *K* = 5. Figure 8A shows the word distributions θ. In the action category, the robot confused the words “reach” and “touch” as is the case with the simulator experiment. Figure 8B shows the learning result of the position category on the table. Figure 8C shows categorization results of objects. Although the object categorization contained a few mistakes, the results were mostly correct. Figure 8D shows the categorization results obtained for the color categorization, which was successful. Interestingly, two categories corresponding to the word “green” were created because the robot distinguished between bright green and dark green. In addition, the robot was able to learn that both of these categories related to the word “green.”

Table 4 shows the evaluation values of the experimental results for 25 and 40 trials. There was not much difference in ARI values between the methods and between different conditions of ROW values. The EAR values of the proposed method were higher than those of the other methods. An increase in the number of trials led to an increase in the evaluation values, similar to the simulation results.

**Table 4:** Experimental results of the CSL task for 25 and 40 trials.

Method	ROW	ARI_a	ARI_p	ARI_o	ARI_c	EAR_*F_d_*
**(A) 25 trials**						
Proposed	0	0.239	0.932	0.201	0.720	**0.866**
w/o MEC-II	0	0.299	0.971	0.207	0.717	0.723
mMLDA	0	0.255	0.959	0.226	0.703	–
Proposed	30	0.297	0.879	0.227	0.702	**0.751**
w/o MEC-II	30	0.242	0.980	0.218	0.683	0.601
mMLDA	30	0.296	0.893	0.256	0.730	–
Proposed	50	0.240	0.905	0.257	0.681	0.604
w/o MEC-II	50	0.224	0.895	0.211	0.694	0.482
mMLDA	50	0.221	0.981	0.303	0.688	–
**(B) 40 trials**						
Proposed	0	0.304	0.960	0.240	0.691	**0.988**
w/o MEC-II	0	0.282	0.986	0.190	0.729	0.763
mMLDA	0	0.290	0.959	0.224	0.736	–
Proposed	30	0.303	0.978	0.193	0.698	**0.829**
w/o MEC-II	30	0.349	0.917	0.219	0.726	0.718
mMLDA	30	0.307	0.956	0.159	0.717	–
Proposed	50	0.316	0.922	0.199	0.718	0.668
w/o MEC-II	50	0.258	0.937	0.210	0.726	0.639
mMLDA	50	0.297	0.989	0.123	0.687	–

*Bold and underscore indicate the highest evaluation values, and bold indicates the second highest evaluation values*.

### 5.2 Action Generation Task

#### 5.2.1 Conditions

In this experiment, the robot generates the action corresponding to the sentence spoken by the human tutor. The robot uses the learning results of the CSL task in Section 5.1. The experimental procedure is the same as in Section 4.3.1. We evaluated accuracy of object selection (the OAR values) using the CSL results for 25 trials. We performed the action generation task for a total of 12 different test-sentences, each of which comprised four words representing the four sensory-channels. The placement of objects on the table was different in each trial and differed from the placements that were used during the learning trials.

#### 5.2.2 Results

**Figure 9:**
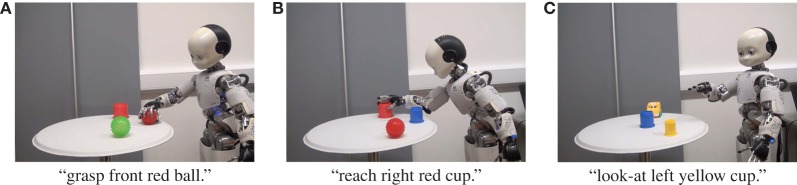
Examples of results of action generation task with real iCub. **(A)** Grasp front red ball. **(B)** Reach right red cup. **(C)** Look-at left yellow cup.

Figure 9 shows an example of the results of the action generation task. Figure 9A shows the result of action generation by the robot for the sentence “grasp front red ball.” Figure 9B shows the result of action generation by the robot for the sentence “reach right red cup.” Figure 9C shows the result of action generation by the robot for the sentence “look-at left yellow cup.” The resulting OAR values of the proposed method and its w/o MEC-II were 1.000, and the OAR value of mMLDA was 0.833. As a result, the robot could select an appropriate object even in situations and sentences not used at the CSL.

### 5.3 Action Description Task

#### 5.3.1 Conditions

In this experiment, the robot performs the action and speaks the sentence regarding this action. The robot uses the learning results of the CSL task in Section 5.1. The experimental procedure is the same as in Section 4.4.1. We use the F1-measure and ACC as evaluation values. We performed the action description task for a total of 10 actions. The placement of objects on the table was different for each trial and differed from those used during the learning trials. The robot generates a sentence of four words representing the four sensory-channels. The word order in the sentence is fixed as *F_d_* = (a, p, c, o).

#### 5.3.2 Results

**Figure 10:**
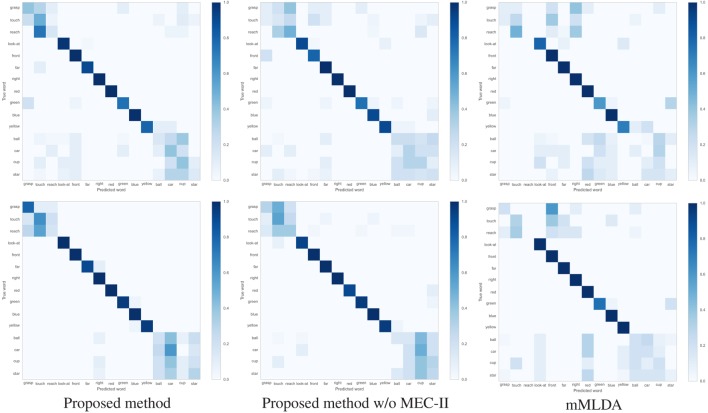
Confusion matrix of results on action description task using the learning result by (top) 20 and (bottom) 40 trials under the ROW is 0%.

Table 5 shows F1-measure and ACC values of action description task using the learning results under the different conditions. The proposed method showed the higher evaluation values than other methods. Figure 10 shows confusion matrices between predicted words and true words using the learning results by 20 and 40 trials. In the action category, there was a tendency to confuse words “reach” and “touch” similar to simulation. The major difference in the result for each method was found in the words of action and object categories. Even if the accuracy of categorization is low as in action and object categories and the categories include uncertainty, the robot could describe the action more correctly if the correspondence between the word and the sensory-channel was performed more properly.

**Table 5:** Experimental results in 25 and 40 trials.

Method	Trials	ROW	F1	ACC
Proposed	25	0	**0.575**	**0.650**
w/o MEC-II	25	0	**0.558**	**0.640**
mMLDA	25	0	0.406	0.558
Proposed	40	0	**0.618**	**0.720**
w/o MEC-II	40	0	**0.654**	**0.698**
mMLDA	40	0	0.509	0.645

*Bold and underscore indicate the highest evaluation values, and bold indicates the second highest evaluation values*.

## 6 Conclusion

In this paper, we have proposed a Bayesian generative model that can estimate multiple categories and the relationships between words and multiple sensory-channels. We performed experiments of cross-situational learning using the simulator and real iCub robot in complex situations. The experimental results showed that it is possible for a robot to learn the combination between a sensory-channel and a word from their co-occurrence in complex situations. The proposed method could learn word meanings from uncertain sentences, i.e., the sentence including four words or less with a changing order. In comparative experiments, we showed that the mutual exclusivity constraint is effective in the lexical acquisition by CSL. In addition, we performed experiments of action generation task and action description task by the robot that learned word meanings. The action generation task confirmed that the robot could also select an object successfully and generate an action even for situations other than those it encountered during the learning scenario. The action description task confirmed that the robot was able to use the learned word meanings to explain the current situation.

The accuracy of the categorization of objects and actions tended to be lower than those of the color and position categories. In this paper, we used GMM for the categorization of each sensory-channel. MLDA achieved highly accurate object categorization by integrating multimodal information (Nakamura et al., 2011a). The accuracy of object categorization can be improved by using MLDA instead of GMM, i.e., by increasing the number of sensory-channels for the object categories. In the action categorization, the robot confused “reach” and “touch,” because these are similar actions. However, the robot is able to classify diverse actions more accurately. In addition, we used static features as action information. The accuracy can be improved by segmenting the time-series data of the actions by using a method based on the hidden Markov model (HMM) (Sugiura et al., 2011; Nakamura et al., 2016).

In this study, we performed the action generation task by a sentence including four words corresponding to the four sensory-channels. However, action instruction also presented cases in which an uttered sentence contains uncertainty. In future, we plan to investigate what kind of action the robot performs based on uncertain utterances, such as when the number of words is fewer than four, when the same objects exist on the table, and when the sentence contains the wrong word. If the robot can learn the word meanings more accurately, the robot would be able to perform an action successfully even from an utterance including uncertainty. Detailed and quantitative evaluation of such advanced action generation tasks is a subject for future work.

Other factors we aim to address in future studies are grammatical information, which was not considered in the present study, and sentences containing five words or more. We showed that the robot could accurately learn word meanings without considering grammar in the scenario of this study. However, it is important to include even more complicated situations with more natural sentences such as “grasp the red box beside the green cup.” More complicated sentences would require us to consider a method that takes the grammatical structure into account. We, therefore, aim to extend the proposed method to more complicated situations and natural sentences. Attamimi et al. (2016) used HMM for the estimation of transition probabilities between words based on concepts, as a post-processing step of mMLDA. However, they were unable to use grammatical information to learn the relationships between words and categories. Hinaut et al. (2014) proposed a method based on recurrent neural networks for learning grammatical constructions by interacting with humans, which is related to the study of an autobiographical memory reasoning system (Pointeau et al., 2014). Integrating such methods with the proposed method may be effective for action generation and action description using more complicated sentences.

In this paper, we focused on mutual exclusivity of words indicating categories in language acquisition. However, there are hierarchies of categories, e.g., ball and doll belong to the toy category. Griffiths et al. (2003) proposed a hierarchical LDA (hLDA), which is a hierarchical clustering method based on a Bayesian generative model, and it was applied to objects (Ando et al., 2013) and places (Hagiwara et al., 2016). We consider the possibility of applying hLDA to the proposed method for hierarchical categorization of sensory-channels.

For future work, we also plan to demonstrate the effectiveness of the proposed method by employing a long-term experiment that uses a larger number of objects. We believe that the robot can learn more categories and word meanings based on more experience. In addition, as a further extension of the proposed method, we intend increasing the types of sensory-channels, adding a positional relationship between objects, and identifying words that are not related to sensory-channels. For example, Aly et al. (2017) learned object categories and spatial prepositions by using a model similar to the proposed model. It would be possible to merge the proposed method with this model in the theoretical framework of the Bayesian generative model. This combined model is expected to enable the robot to learn many different word meanings from situations more complicated than the scenario in this study.

## Author Contributions

AT, TT, and AC conceived, designed the research, and wrote the paper. AT performed the experiment and analyzed the data.

## Conflict of Interest Statement

The authors declare that the research was conducted in the absence of any commercial or financial relationships that could be construed as a potential conflict of interest.

## Supplementary Material

Video S1 Cross-situational learning scenario using a real iCub.

Video S2 Action generation task and action description task using a real iCub.
